# Digital Health Interventions Incorporating Behavior Change Techniques and Gamification for Bladder Health in Adults Aged 50 Years and Older: Rapid Review

**DOI:** 10.2196/76867

**Published:** 2026-08-26

**Authors:** Oscar Aguila-Gimeno, Norina Gasteiger, Emma Stanmore, Marius Brazaitis, Rima Solianik, Erika Karkauskiene, Laura Jarutienė, Júlia Romeu-Busquets, Javier Jerez-Roig

**Affiliations:** 1 Faculty of Health Sciences and Welfare, Research Group on Methodology, Methods, Models and Outcomes of Health and Social Sciences (M_3_O), Centre for Health and Social Care Research (CESS). University of Vic-Central University of Catalonia (UVic-UCC), Vic, Spain; 2 Institute for Research and Innovation in Life Sciences and Health in Central Catalonia (IRIS-CC), Vic, Spain; 3 Hospital Universitari de Santa Maria, Lleida, Spain; 4 School of Health Sciences, The University of Manchester, Oxford Rd, Manchester, Manchester, M13 9PL, United Kingdom, 44 161 306 600; 5 Institute of Sport Science and Innovations, Lithuanian Sports University, Kaunas, Lithuania, Lithuania

**Keywords:** urology, gynaecology, urinary incontinence, pelvic floor exercises, pelvic floor muscle training, behavior change, digital health, healthy aging, gamification

## Abstract

**Background:**

Urinary incontinence (UI) is the most prevalent pelvic floor dysfunction, with incidence increasing with age. Numerous studies have demonstrated the effectiveness of pelvic floor exercises in improving UI. However, access to pelvic floor treatment remains limited due to lengthy waiting lists and poor adherence to prescribed exercises. Digital solutions incorporating behavior change techniques (BCTs), including gamification elements, may support self-management of bladder health for individuals aged 50 years and older who may face challenges in accessing conventional treatments.

**Objective:**

This study aimed to identify mobile apps and websites integrating BCTs, including gamification elements, to facilitate bladder health self-management among adults aged 50 years and older.

**Methods:**

The search was initiated in July 2024 and updated in January 2025 across three sources: (1) mobile app stores (Google Play Store and Apple App Store available in Spain, Lithuania, and the United Kingdom) using the keywords “pelvic floor,” “urinary incontinence,” and “bladder”; (2) websites; and (3) academic databases (PubMed, Scopus, CINAHL, and Google Scholar) for journal articles, book chapters, and conference papers published in English, Spanish, or Lithuanian. Inclusion criteria required that solutions be independently usable without health care supervision, incorporate at least one BCT (eg, training, education), be evidence-informed (ie, include participants aged ≥50 years in their design or piloting phase or explicitly target this demographic), and be available in Spain, the United Kingdom, or Lithuania. The Mobile App Rating Scale (MARS) was used to assess app quality, and the taxonomy of 93 BCTs was used for BCT classification. All review and data extraction processes were conducted in duplicate.

**Results:**

Twenty-one studies met the inclusion criteria, identifying 8 eligible mobile apps and 1 website. Among the apps, only 2 were available on either Google Play or the Apple App Store. No apps were identified in Lithuania, whereas 1 app was found in Spain and 3 apps in the United Kingdom. Of these, 1 UK app was accessible on both Google Play and the Apple App Store, whereas the others were limited to a single platform. BCT extraction showed that the apps included between 9 and 16 BCTs (mean 12, SD 3.27). Regarding quality, all assessed apps obtained MARS scores ranging from 3 to 4 out of 5. Website searches did not identify any scientifically validated platforms across the 3 countries, except for 1 website cited in a scientific publication. UI reduction on the International Consultation on Incontinence Questionnaire ranged from –3.9 to –2.1 points, while perceived improvement reached 91.7% in the Tät app.

**Conclusions:**

Evidence-based digital interventions for individuals aged 50 years and older remain limited. Existing apps suggest potential benefits in UI reduction and quality of life improvement; however, further research and development are needed to enhance accessibility and efficacy.

## Introduction

The incidence of pelvic floor dysfunction is increasing, especially among older adults. Urinary incontinence (UI) is the most common dysfunction of the pelvic floor, and the incidence increases with age. UI is the involuntary loss of urine, affecting up to 36 million people in Europe [1-3].

The total direct cost in Germany, Italy, Spain, Sweden, and the United Kingdom has been estimated at €5 billion per year (€1=US $1.14 as of July 12, 2026), and this burden will increase with the projected 25% increase in UI prevalence by 2030 [2]. The mean annual per capita cost of its treatment is €300‐€600 per country [2].

The most frequent types of UI are stress urinary incontinence, which is characterized by the involuntary leakage of urine during activities that increase intraabdominal pressure, such as coughing or sneezing; urgency urinary incontinence, defined by a sudden and compelling need to urinate, usually accompanied by leakage before reaching the toilet; and mixed urinary incontinence, which combines features of both previous types [4].

Several studies have explored the effectiveness of pelvic floor exercises to improve UI and reduce the involuntary loss of urine [5,6]. A combination of behavioral therapies consisting of pelvic floor muscle training, bladder training, and practical tips is beneficial in the management of UI [5]. Also, several studies have indicated that low levels of physical activity (PA) and prolonged patterns of sedentary behavior represent risk factors for UI [7,8], with PA being defined by the World Health Organization as any bodily movement produced by skeletal muscles that requires energy expenditure [9]. Some individuals with UI cannot access pelvic floor treatment because there are barriers to uptake, including waiting lists that limit access to treatment, poor adherence to exercises [1,10], the price of the treatment, or embarrassment.

Ultimately, pelvic floor dysfunctions can reduce the quality of life of older people, with research showing that several interrelated factors can challenge the independence of older adults: primarily functional and cognitive impairment, chronic diseases, a diminishing social network, and a low level of PA [11].

Gamification should not be understood merely as the incorporation of game elements into nongame contexts, but rather as a process aimed at generating meaningful psychological experiences that enhance the perceived value for the user. This emphasizes that motivational affordances (such as points, levels, or badges) function in nongame contexts as stimuli designed to activate psychological needs and emotional states, with the purpose of influencing behavior and strengthening emotional engagement [12].

In contrast to gamification, video games constitute complete, self-contained interactive environments designed primarily for entertainment. While video games involve full game narratives, mechanics, and immersive worlds, gamification selectively integrates specific game design elements into health or behavioral interventions without necessarily creating a full game experience. This conceptual distinction is essential, as gamified interventions and video-game–based interventions may rely on different psychological mechanisms, levels of immersion, and theoretical foundations [13,14].

Grounded in motivational psychology, gamification fosters constructs such as self-efficacy, engagement, and habit formation, particularly through the promotion of autonomy, competence, and social relatedness [13]. These factors are directly linked to self-determination theory, which highlights the importance of satisfying basic psychological needs in order to sustain intrinsic motivation [13].

Furthermore, gamification is conceived as an attempt to increase the likelihood of the emergence of experiences of flow, challenge, or immersion [14], rather than as a fixed set of game mechanics. In this way, psychology becomes the central axis explaining how design elements are transformed into emotional and motivational experiences, which ultimately determine the effectiveness of gamification in creating value for the user [12].

In recent years, emerging evidence suggests that gamification may serve as a valuable tool in addressing health-related challenges among patients [15]. According to the meta-analysis conducted by Mazeas et al [15], gamified interventions are not only effective in modifying behavior, but also demonstrate greater efficacy compared to other behavioral approaches. Moreover, these interventions show promise in supporting the long-term maintenance of behavioral changes.

Another review highlights that supervised exercise programs incorporating game elements appear to increase PA levels more effectively than conventional exercise routines [16].

Behavior change techniques (BCTs) are defined as observable, replicable, and irreducible components of an intervention designed to modify behavior [17]. They represent the smallest active ingredients that can be applied individually or in combination to influence behavioral outcomes. BCTs, such as feedback or monitoring, can enhance the maintenance of behavior over time through instant feedback and improve performance in eHealth programs [18]. Behavioral treatment for pelvic floor dysfunctions can be an effective tool either on its own or in combination with medication to improve UI, urinary urgency, nocturia, and other conditions [19].

In the context of mobile technologies and web-based interventions targeting bladder and pelvic floor health, the application of the BCT taxonomy alongside gamification enables the identification of active ingredients that promote adherence, engagement, and sustained behavior change in adults and older populations.

Given the growing number of pelvic floor apps available online, this review focused on evidence-based programs to assess their actual effectiveness. A 2019 systematic review identified 840 pelvic floor–related apps, of which approximately 78% were not developed by healthcare professionals. This raises concerns regarding the usability and clinical efficacy of apps that lack a foundation in scientific evidence [20].

Rapid reviews have been recognized by the Cochrane Rapid Reviews Methods Group and the World Health Organization as an appropriate approach when timely evidence is needed to inform intervention development in fast-evolving fields [21,22]. In the context of digital health for bladder and pelvic floor self-management, technologies evolve quickly and new apps emerge each year, making a rapid review particularly suitable for providing up-to-date, actionable insights. To ensure methodological rigor despite the accelerated timeline, we followed established rapid review guidance, including the use of predefined eligibility criteria, structured search strategies, and transparent reporting of methodological shortcuts [21]

Technology can offer a solution to some of these challenges, such as UI. The willingness of older people to use technology depends on their perceived need. Initially, older people may believe that technology can be useful for others, but not for them. However, when they try it, they tend to want to continue using it [23]. Due to the increasing prevalence of pelvic floor disorders and the growing importance of technology in health and daily life, it is important to examine mobile technologies and websites designed for self-management among these populations. Therefore, the specific objective of this work was to synthesize the current literature and available apps in Spain, Lithuania, and the United Kingdom for the promotion of bladder health and/or the management of UI among adults aged 50 years and older.

## Methods

### Overview

This review is the first part of the KOKU Bladder project (ClinicalTrials.gov, NCT06583733), an international collaboration involving the University of Vic (Spain), Lithuanian Sports University (Kaunas, Lithuania), and the University of Manchester (United Kingdom). The review included mobile apps, websites, and academic databases from each participating country. Where possible, we adhered to the recommended 7-step method for conducting reviews of mobile health apps [24]. We used a similar approach to combining reviews of the evidence, smartphone apps, and websites as that used in a review conducted by McGarrigle et al [25]. The protocol was registered on October 4, 2024, in PROSPERO (reference CRD42024597624). The Population, Intervention, Comparison, and Outcome (PICO) framework was used to guide the formulation of the inclusion criteria. Pilot searches were conducted in July 2024 to identify appropriate search keywords, followed by the final search between October 2024 and January 2025.

The rapid review was conducted to preliminarily explore the available literature to lay the foundation for the project and guide the initial stages of work. To achieve this, a targeted search methodology was applied, examining a smaller number of databases compared to a traditional systematic review, which allowed for a faster process. In addition to databases, websites and app stores were also reviewed. The total duration of the study was four months, a shorter period than that required for more exhaustive reviews, but sufficient to obtain an overview of the existing evidence and support the project’s initial decision-making.

### Eligibility Criteria

The following criteria were used to select relevant digital solutions (apps or websites) across the three searches (Textbox 1).

Textbox 1. **Inclusion and exclusion criteria.**

**Inclusion criteria**:

- Apps or websites that can be used by people independently, without supervision by a healthcare professional.
- Digital technologies (apps and websites) regardless of cost (eg, to download or to access in-app functions).
- Interventions including at least one behavior change technique (eg, training, education, monitoring, and prompting).
- Evidence-informed: resources appropriate for use with older populations (ie, they have included participants aged >50 years in the design or piloting phase or target this group). They do not have to exclusively include those aged >50 years, but rather be part of the study group.
- Available in Spain, the United Kingdom, or Lithuania and in English, Spanish, or Lithuanian.

**Exclusion criteria**:

- Apps or websites designed for pregnant or postpartum individuals, those aged <50 years, or health and social care professionals.
- Apps or websites only including pelvic floor muscle techniques and/or training.
- Apps or websites that require invasive devices (eg, intravaginal devices).
- Intended only for health care professionals.
- For the academic database searches, we excluded review studies, case reports, letters to the editor, editorials, conference abstracts, and personal opinions and/or commentaries.

### Search and Screening

The entire review process was conducted by at least 2 researchers in each participating country. The search and retrieval of scientific papers were carried out by the Spanish research team (OA-G and JJ-R) in October 2024. All retrieved papers were subsequently uploaded to the Rayyan (Rayyan Systems Inc.) bibliographic management platform for review by the research team [26].

At the outset of the review process, duplicate records were identified and removed using Rayyan’s automated detection system. Following this, the titles and abstracts of all papers were screened by the research team, ensuring that each paper was reviewed by a minimum of 2 independent reviewers. The selected papers were then downloaded, thoroughly read, and subjected to screening in duplicate. The initial review was conducted by 1 researcher from Spain (OA-G) and 1 from the United Kingdom (ES). A secondary review was carried out by the Lithuanian team (RS, EK, and LJ), comprising three researchers. Upon completion of this phase, a second Spanish researcher examined and resolved any conflicts (JJ-R), leading to the final selection of papers. The extracted data were subsequently organized into tables for analysis.

For the search of mobile apps and websites, at least 2 researchers conducted the process in each country: OA-G, JJ-R, and JRB in Spain; ES and NG in the United Kingdom; and RS, EK, and LJ in Lithuania. The search results were compiled and recorded in tables. Subsequently, OA-G and NG reviewed the findings, while JJ-R resolved any discrepancies or uncertainties.

### App Market

A systematic search was conducted in the Google Play Store and Apple App Store in Spain, Lithuania, and the United Kingdom. Searches were performed on Android (Samsung Galaxy Note 20 Ultra, Android 11; Samsung Galaxy S24, Android 14 OS) and iPhone (iPhone 12, iOS 16.1.1). The search keywords included “pelvic floor,” “urinary incontinence,” and “bladder.” Equivalent terms in Spanish and Lithuanian were used for searches within the respective app markets.

A peer review process was implemented for the app search to ensure consistency and accuracy. The final results from all 3 countries were compiled and compared in a comprehensive data table.

### Websites

We searched for websites featuring interventions aimed at promoting behavior change related to bladder health (eg, UI) with supporting scientific evidence. The search was conducted in 3 countries and 3 languages, with each country searching in its native language. The search strategy was in English included the terms UI exercises, pelvic floor exercises, pelvic floor muscle training, and bladder exercises. In Spanish: ejercicios para la incontinencia urinaria, ejercicios de suelo pélvico, entrenamiento muscular del suelo pélvico, and ejercicios de vejiga. In Lithuanian: šlapimo nelaikymo pratimai, dubens dugno pratimai, dubens dugno raumenų treniravimas, and šlapimo pūslės pratimai.

The search was conducted using Google. We systematically reviewed the first 20 search results from each country for each keyword. This cutoff was based on user behavior research indicating that most users only explore the first few links in search engine results [27]. Rew et al [28] reported that the number of websites reviewed per keyword varies from 10 to 200, with the first 25 websites being a commonly used number.

All the websites identified were reviewed to determine whether they included scientific papers supporting the effectiveness of the site in reducing UI.

### Academic Databases

A systematic search was conducted to identify scientific evidence supporting the included mobile apps and websites. The search was performed in the following academic databases: PubMed, Scopus, CINAHL, and Google Scholar.

Eligible sources included journal papers, book chapters, and full-length conference papers published in English, Spanish, or Lithuanian.

The search strategy in PubMed was ((“pelvic floor”) OR (urinary incontinence) OR (bladder)) AND ((app*) OR (mHealth) OR (eHealth) OR (game*)), in SCOPUS we limited to paper and aged, and the search strategy was: ((“pelvic floor”) OR (“urinary incontinence”)) ((app*) OR (mhealth) OR (ehealth) OR (technology)). In CINAHL we limited by source type (academic journal) and age (aged 80 years and older and middle-aged: 45‐64 years years), and the search strategy was ((“pelvic floor”) OR urinary incontinence OR bladder) AND (app* OR mHealth OR eHealth OR game*)

### Evaluation, Data Extraction, and Synthesis

#### Evaluation: Mobile App Rating Scale

The quality of the apps was evaluated using the validated Mobile App Rating Scale (MARS) [29] by 2 researchers in the United Kingdom (ES and NG) and in Spain (OA-G and JR). The criteria assess quality by considering engagement, functionality, esthetics, information quality, and subjective quality, leading to the development of 23 subcategories from which the 23 individual MARS [29] items are derived. Each item is rated on a 5-point scale (1=inadequate, 2=poor, 3=acceptable, 4=good, and 5=excellent), with descriptors provided for each rating anchor. In cases where an item was not applicable, a “not applicable” option was included. In this review, we used the first 19 subcategories and excluded the last category, “subjective quality.”

MARS [29] was used in duplicate, and we calculated the mean scores for the final results for each app.

#### Evaluation: BCTs

To analyze the BCTs in the apps, we used the taxonomy of BCTs developed by Michie et al [30]. The taxonomy includes a list of 93 BCTs, each evaluated with a score of “0” (absent) or “1” (present). Scores were then summed. The total score for each app ranged from 0 to 93, with higher scores reflecting a greater implementation of BCTs [30].

BCTs were also determined in duplicate, in the United Kingdom (ES and NG) and in Spain (OA-G and JR). Consensus was reached for any apps in which there was disagreement between the researchers.

#### Evaluation: Databases

During the review process, data from the included studies were extracted systematically into predefined tables. For each intervention study, we recorded key sample characteristics such as mean age, number of participants, educational level, and type of UI, as well as the assessment tools used and the main conclusions reported. This approach allowed us to compare study populations and to examine differences across the validated questionnaires used.

For the included studies, we collected demographic and clinical variables (sample size, age, education level, percentage of women, and UI type) to contextualize the populations targeted by each intervention. These characteristics are essential for assessing the applicability of digital tools to older adults, who often present heterogeneous clinical profiles and varying levels of digital literacy. In addition, we extracted the validated questionnaires used in each study (eg, International Consultation on Incontinence Questionnaire - Short Form (ICIQ-SF), International Consultation on Incontinence Questionnaire - Overactive Bladder (ICIQ-OAB), Patient Global Impression of Improvement (PGI-I), and Pelvic Muscle Physical Therapy (PMPT)), as these instruments represent the most widely accepted clinical and patient-reported outcomes in UI research.

Once the final set of eligible papers was identified, we conducted a structured data extraction process. All studies were read in full, and the relevant information was systematically recorded in predefined tables. For each intervention study, we extracted demographic and clinical characteristics of the sample, including mean age, number of participants, education level, and type of UI, as well as details on study design and the assessment instruments used.

Once the final set of eligible papers was confirmed, we refined the extraction through a structured, full-text review of each study. In parallel, information on the identified mobile apps was collected by analyzing both the app content and the associated scientific publications. App-related data included platform availability, version number, cost, country of origin, and study setting. BCTs were identified using the Behavior Change Technique Taxonomy, app quality was assessed using the MARS [29], and gamification elements were identified through direct inspection of the apps, noting features such as levels, progress tracking, rewards, or visual feedback. All extracted information was organized to ensure consistency and facilitate comparison across studies and apps.

The results were collected in 3 tables. Table 1 contains data related to the apps, the country in which they were found, app store, version number, developer name, price, study setting, number of BCTs, and MARS [29] outcome (Multimedia Appendix 1). Table 2 includes the general information for the selected papers: app name, sample size, mean age, educational level, percentage of women, study design, and percentage of IU (Multimedia Appendix 2). Finally, Table 3 provides the results of the papers and the different questionnaires (Multimedia Appendix 3).

**Table 1.** Technological and qualitative information of the apps.

Name of app	Country	App market	Version number	Developer name	Cost	Study setting	BCTs^a^	MARS^b^
BladderBoss	United Kingdom	Apple App Store	1.8	Amara Therapeutics	First week free, after monthly €24.99	Ireland	16	Good 4 (0.20)
Tät app	Spain	Google Play	5.2.7	eContinence AB	First week free, after €3.19 per month	Sweden	9	Acceptable 3.39 (0.02)
BladderBoss	United Kingdom	Google Play	1.0.443	Amara Therapeutics	First week free, after monthly €24.99	Ireland	17	Acceptable 3.35 (0.05)
URinControl	Spain	Google Play	1.2.5	Curavista	Free	Sweden and Netherlands	10	Acceptable 3.33 (0.07)
URinControl	United Kingdom	Google Play	1.2.5	Curavista	Free	Sweden and Netherlands	9	Acceptable 3.33 (0.07)
URinControl4all	United Kingdom	Apple Store	1.0.0	Curavista	Free	Netherlands	11	Acceptable 3.0 (0.20)

a BCT: behavior change technique.

b MARS: Mobile App Rating Scale.

**Table 2.** General information of the papers.

Reference	Name of app or webpage	Sample size (control/ intervention group), % (n/N)	Mean age (range or SD)	Education level (%, university or higher)	Women (%, n/N)	Study design	IU^a^ types (%, n/N)
[31]	Tät II	123 (60/63)	58.3 (31-77)	Majority	100 (123/123)	RCT^b^	MUI:^c^ 72 (89/123) and UUI:^d^ 22.80 (28/123)
[32]	Tät II	122 (60/62)	58.3 (SD 9.6)	NR^e^	100 (122/122)	RCT (cost-utility analysis)	NR
[33]	Tät II	123 (60/63)	58.6 (49.5‐67.7)	61.8 (76/123)	100 (123/123)	RCT	MUI: 73.5 (90/123) and IUU: 26.5 (33/123)
[34]	Tät II	123 (61/62)	(27-72) Responders at 2 years (mean age 44.2±10.3), and non-responders at 2 years (mean age 46.4, SD 7.8)	NR	100 (123/123)	Prospective cohort RCT	SUI:^f^ 100 (123/123)
[35]	URinControl	262 (131/131)	53 (20-86)	NR	100 (262/262)	RCT	MUI: 50 (131/262)
[36]	URinControl	9	(32-68)	NR	100 (9/9)	Qualitative	SUI: 11 (1/9), UUI: 33 (3/9), and MUI: 56 (5/9)
[37]	URinControl	17	(35-78)	NR	100 (17/17)	Mixed methods	NR
[38]	URinControl	262 (131/131)	(mean 52.2, SD 11.6)	52.7 (138/262)	100 (262/262)	Prediction model based RCT	SUI: 68.7 (180/262) and UUI: 31.3 (82/262)
[39]	URinControl	262 (131/131)	54 (23‐86)	NR	100 (262/262)	RCT	SUI: 34 (89/262)
[40]	Tät	2672 (0/2672)	43.7	65.3 (1745/2672)	100 (2672/2672)	Cohorts study	SUI: 53.1 (1419/2672), UUI: 12.1 (232/2672), and MUI: 30.9 (1021/2672)
[41]	Tät	15	47 (27‐72)	NR	100 (15/15)	Qualitative Interview	NR
[42]	Tät	123 (61/62)	44.7 (SD 9.4)	NR	100 (123/123)	Cost-utility analysis of a previous RCT	SUI: 100 (123/123)
[43]	Tät	61	45 (27‐72)	90.2 (55/61)	100 (61/61)	Secondary analysis of RCT treatment G	SUI: 100 (61/61)
[44]	Tät	98	58 (37‐77)	86.0 (84/98)	100 (98/98)	Secondary data analysis of RCT	MUI: 74 (72/98) and UUI: 26 (22/98)
[45]	Tät	123 (61/62)	44.7 (27-72)	80 (98/123)	100 (123/123)	RCT	SUI
[46]	MiHealth Bladder	29 (0/29)	54.4 (29-77)	55 (16/29) university	100 (29/29)	Single group pretest or posttest design	NR
[47]	Diário Saúde	21 initials (9/12)	App group 47.2±10.06 control group 53.3±13.2	NR	100 (21/21)	RCT	SUI
[48]	Diário Saúde	156	49.3±14.2	NR	100 (156/156)	RCT - single group	SUI
[49]	Yoga of Immortals YOI	422	(18 - 74)	NR	94.5 (399/422)	Prospective cohort	NR
[50]	Web page: www.tät.nu [51]	275 (booklet 109 and internet 166)	booklet group 59.4 years Internet group 54.5 years	59 booklet group, (64/109) and 67 internet group (111/166)	100 (275/275)	Pragmatic prospective cohort	NR
[52]	iDry Usage Results of a Mobile App for Managing UI^g^	878	51 (SD 17.6).	NR	25.4 (223/878)	Obser vational	NR

a UI: urinary incontinence

b RCT: randomized controlled trial.

c MUI: mixed urinary incontinence.

d UUI: urgency urinary incontinence.

e NR: no results.

f SUI: stress urinary incontinence.

g UI: urinary incontinence.

**Table 3.** Conclusion of the papers. Incontinence episode frequency was calculated per bladder diary entries.

Reference	ICIQ-SF	ICIQ-OB	ICIQ-LUTSqol	PGI-I	Patient satisfaction	App usage	PFMT^a^	Interview	UI^b^ frequency*	Other instruments	Main results of IG^c^
[31]	✓	✓	✓	✓	✓				✓	Incontinence Catastrophizing Scale	Improvement in all parameters
[32]	✓		✓							Costs for assessment, treatment delivery, incontinence aids, laundry, time for PFMT and BT^d^, QALYs^e^, and ICER^f^	Higher quality of life and annual cost (€738.42 vs €605.82).
[33]	✓	✓	✓	✓	✓					—	Reduction in the severity of UI, improvement of OB^g^ symptoms, and QoL.^h^
[34]	✓	✓				✓	✓			—	Long-term efficacy, achieving a significant reduction in urinary symptoms, similar in both groups.
[35]	✓		✓	✓					✓	—	Long-term efficacy, achieving a significant reduction in urinary symptoms, similar in both groups.
[36]	✓									3IQ, eHEALS^i^, TAM, and MARS^j^	The app could facilitate access to treatment, increase self-awareness, and improve adherence.
[37]								✓		—	Treatment success was influenced by factors such as adherence, personality, exercise integration, and prior experience, while the main barriers were unrealistic expectations and personal difficulties.
[38]	✓							✓		Prognostic factors and modifiers were combined	Personal factors such as symptom severity, age, educational level, and impact on quality of life are crucial in predicting the effectiveness of eHealth treatments for UI.
[39]	✓		✓							EQ‐5D‐5L	Improvements in the severity of UI for at least one year, with no significant differences between groups.
[40]	✓			✓		✓				—	APP group: significant reduction in the severity of UI and improvement in its symptoms.
[41]						✓	✓	✓		—	Using the app empowered women and helped them self-manage their UI treatment by motivating them to follow a PFMT.
[42]	✓		✓			✓	✓		✓	CTA^k^, training time, UI assistive devices, and laundry	The app is cost-effective for the treatment of UI. The annual cost was €547.00 app group and €482.40 control group. Greater gains in life years were observed at a similar cost.
[43]	✓		✓	✓			✓			—	Treatment expectations, weight change during treatment, and self-reported PFMS^l^ muscle strength were significantly associated with successful SUI^m^ treatment outcome using an app.
[44]	✓	✓	✓	✓		✓	✓		✓	—	Participants were satisfied with the treatment 15 months after having access to the app
[45]	✓		✓	✓			✓			IEF	IG: improvements in symptom severity, QoL, and PGI-I scores.
[46]	✓			✓	✓		✓			—	IG: It is effective in reducing UI throughout the intervention
[47]	✓									sEMG and PFM examination; ICIQ-VS; QUID. App usage. Oxford Modified Scale	Improvements in symptoms and QoL were observed, with no significant differences between groups.
[48]	✓	✓								QUID-Portuguese	App treatment was highly effective in improving QoL and UI
[49]	✓		✓	✓						—	Improvement in UI severity, QoL, and PGI-I scale at 4 and 8 weeks
[50]	✓									—	UI improvement in both groups with no significant differences
[52]	✓	✓	✓	✓						—	Improvements in reducing pad use, with a 20% decrease in daily use in the short term.

a PFMT: pelvic floor muscle training.

b UI: urinary incontinence.

c IG: intervention group.

d BT: Bladder Training

e QALY: quality-adjusted life-year.

f ICER: incremental cost-effectiveness ratio.

g OB: overactive bladder.

h QoL: quality of life.

i eHEALS: eHealth Literacy Scale.

j MARS: Mobile App Rating Scale.

k CTA: costs for treatment administration.

l PFMS: pelvic floor muscle strength.

m SUI: stress urinary incontinence.

The selection of outcomes in this review was guided by the primary objective of identifying mobile apps and websites that integrate BCTs and gamification elements to support bladder health self-management among adults aged 50 years and older. Accordingly, we prioritized outcomes that capture both the behavioral components of the interventions and their practical relevance for end users. For the included apps, we extracted information on cost, study setting, presence of BCTs, MARS scores [29], country of origin, and app-store availability, as these indicators reflect accessibility, design quality, and behavioral content, which are key dimensions in evaluating digital self-management tools for older adults.

No specific methods for data preparation or synthesis, such as handling of missing summary statistics or data conversions, were required.

No additional analyses were conducted to explore heterogeneity (eg, subgroup analysis or meta-regression), nor were sensitivity analyses performed to assess the robustness of the synthesized results. Likewise, no specific methods were applied to evaluate risk of bias due to missing results or reporting biases, and no formal assessment of certainty or confidence in the body of evidence was undertaken. These procedures were not applicable to our study.

## Results

### Overview

**Figure 1.**
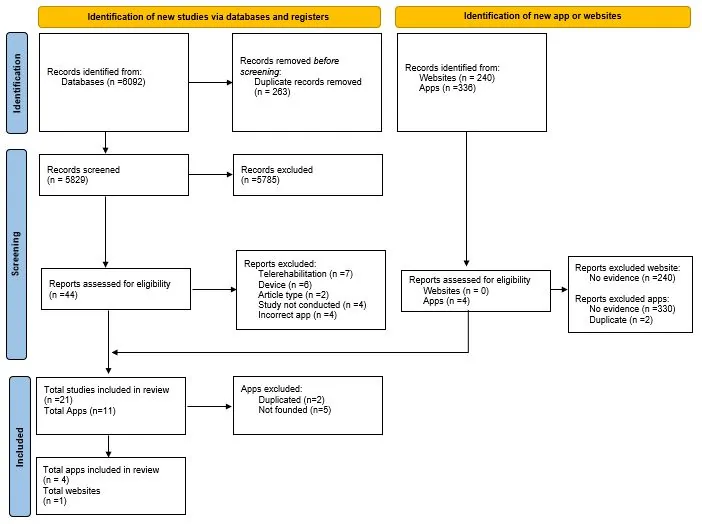
Flowchart depicting the search and screening process.

Following the screening process, 21 papers were deemed eligible for inclusion. These papers cover 7 distinct mobile apps and 1 website. The entire search process is illustrated in Figure 1 (flowchart).

### Database Results

From the academic database searches, we identified seven apps with scientific evidence; however, we were only able to download two of them, Tät (eContinence AB) and URinControll (Curavista). The apps that we could not download or locate were Tät II (Curavista), MyHealtheBladder (U.S. Department of Veterans Affairs), Diário Saúde (Eliza Ferrari), Yoga of Immortals–YOI (Ishan Shivanand (originating from ShivYog), and iDry (Ruslan Pavliuchenko) apps. Although these apps have scientific evidence, they were unavailable for download from Google Play or the Apple App Store.

For the app search, no apps were found in Lithuania. In Spain, we identified only 1 app (URinControl; Curavista), while in the United Kingdom, we found 3 different apps (URinControl, URinControl4all, (Curavista) and BladderBoss (Amara Therapeutics)). BladderBoss was available on both Google Play and the Apple App Store (as different versions), whereas the other apps were available in only one app store. Table 1 presents the technological and qualitative information about the apps.

The website search did not identify any platforms with scientific evidence supporting their effectiveness across the 3 countries. In total, 80 webpages were reviewed in each country, but none met the inclusion criteria. Only 1 website with scientific evidence was found through the scientific paper from the search: the Tät website (eContinence AB) [51].

Following the completion of the search process, a total of 4 mobile apps were identified as having scientific evidence and were available for download via Google Play or the Apple App Store. UrinControl4all should be considered a version of URinControl because it is based on the same scientific paper and differs only by minor variations (eg, language). We extracted data from the final papers and compiled them into Tables 2 and 3 to facilitate comparison and readability.

In the clinical questionnaire results, Tät showed the greatest reduction in UI severity (ICIQ-SF score: –3.9 points) and in symptom-related quality of life measured using the International Consultation on Incontinence Questionnaire Lower Urinary Tract Symptoms Quality of Life. [1](ICIQ-LUTS-QoL score: –4.8 points), together with a perceived improvement of 91.7% according to the PGI-I, indicating a statistically significant improvement. URinControl or URinControl4all showed reductions of –2.16 and –4.34 points, respectively, with a perceived improvement of 65.7%, while BladderBoss assessed questionnaires specific to overactive bladder (ICIQ-OAB score: –2.13 and ICIQ-OAB-QoL score: –22.04 points) and did not report PGI-I data.

### BCT Results

In the BCT extraction, we identified that the apps had between 9 and 16 BCTs (mean 12, SD 3.27). All apps had BCTs related to goals and planning, feedback and monitoring, shaping knowledge, repetition and substitution, and comparison of outcomes. None had BCTs related to social support, regulation, antecedents, identity, scheduled consequences, or covert learning.

Regarding the distribution of BCT categories, the most frequently represented techniques were those associated with goals and planning, feedback and monitoring, shaping knowledge, repetition and substitution, and comparison of outcomes, each appearing six times, meaning that they were present in all the reviewed apps. In contrast, the techniques related to association and comparison of behavior appeared five times. Categories such as natural consequences showed a lower representation, appearing only twice, and the same frequency was observed for reward and threat, and self-belief. No BCTs were identified for social support, antecedents, regulation, identity, scheduled consequences, or covert learning. This pattern indicates that most apps emphasize self‑regulatory and educational strategies rather than social or identity‑based components.

### MARS Results

The MARS evaluation results were obtained through a paired review of the apps. The highest-rated app was BladderBoss (Apple App Store), which achieved a good score of 4.0 (SD 0.20). The remaining apps received scores within the acceptable range. Specifically, Tät (Spain) obtained an acceptable score of 3.39; BladderBoss on Google Play also scored 3.39 (SD 0.02); URinControl received an acceptable score of 3.33 (SD 0.07), with identical ratings in Spain and the United Kingdom; and URinControl4all achieved an acceptable score of 3.0 (SD 0.20).

This rapid review did not include detailed assessments of risk of bias for individual studies or synthesis-level evaluations of bias. Similarly, investigations of heterogeneity (such as subgroup analyses or meta-regression), sensitivity analyses to test robustness, assessments of reporting bias due to missing results, and evaluations of certainty or confidence in the body of evidence were not undertaken. These procedures were not incorporated because they fall outside the scope and objectives of a rapid review, which prioritizes timeliness and streamlined synthesis over comprehensive methodological appraisal [53].

## Discussion

### Principal Findings

A systematic search was conducted to identify mobile apps and websites related to pelvic floor training. A total of 6092 papers, 240 webpages, and 336 apps were peer reviewed, resulting in only 4 apps that were available and met our inclusion criteria. All the apps identified received an “acceptable” rating on the MARS scale [29], and one achieved a “good” rating. Regarding the BCTs, all included between 9 and 17 BCTs, and some also incorporated elements of gamification.

A total of 11 apps were identified as having scientific publications supporting their efficacy. Of these, four apps met the criteria for both scientific validation and mobile availability: URinControl, Tät, URinControl4all, and BladderBoss. All were accessible via the Google Play Store, while BladderBoss was additionally available on the Apple App Store, suggesting a dominance of android-based development in this field. The URinControl and URinControl4all apps are based on the same scientific study to validate their effectiveness. They also have similar content, differing only in language and the way users access the same program. It is important to take this into account, as URinControl4all can currently be supported by the URinControl paper [35-37]; however, if both apps are updated, it is possible that these findings may no longer be applicable.

The search was conducted in Lithuania, the United Kingdom, and Spain. Notably, no relevant apps were identified in Lithuania. In contrast, 2 apps were found in Spain and 4 in the United Kingdom. A possible explanation for this discrepancy is that all identified apps were developed in English-speaking countries, potentially eliminating the necessity for translation into other languages. Indeed, market research conducted by the Organisation for the Review of Care and Health Apps (ORCHA) has reported that of the available 311,000 health apps worldwide, 227,500 (73%) are available in the United Kingdom [54].

Regarding MARS scores [29], all assessed apps scored between 3 and 4 out of 5, indicating acceptable quality. The highest-rated app was BladderBoss (version 1.8 for iOS). The most highly rated section across all apps appears to be the “Information” section, which provides high-quality content such as text, feedback, measurements, and references. However, in some cases, the app description in the App Store and the definition of measurable objectives could be improved. However, it is important to note that app evaluation frameworks do not always reflect real-world engagement [55], with MARS scores showing to have weak correlation with user retention after downloading an app [56]. Additionally, key parameters of interest, including the implementation of gamification, and the resolution of graphics are consistently well-rated across all apps. Conversely, most apps lack sufficient visual appeal and would benefit from a more detailed description of their functionalities.

The presence of BCTs within the apps was also assessed. BladderBoss demonstrated the highest incorporation of BCTs, scoring between 16 and 17 points, depending on the evaluated version. All analyzed apps incorporated at least 7 BCTs, highlighting their potential effectiveness in facilitating behavioral modifications. Notably, all apps included BCTs related to goals and planning, feedback and monitoring, shaping knowledge, repetition and substitution, and comparison of outcomes. These techniques are fundamental in guiding users through behavioral change processes [23]. Furthermore, association and comparison of behavior were present in 5 out of the 6 analyzed apps, indicating a widespread inclusion of these techniques.

Conversely, none of the apps integrated BCTs associated with social support, regulation, antecedents, identity, scheduled consequences, or covert learning. This absence suggests potential areas for improvement in future app development to enhance user engagement and support. A study highlights the significance of behavior change and its influence on the adoption of home-based technology by older adults [11]. Specifically, the study emphasizes how personal perceptions and behavioral factors shape technology use in this population. Given the impact of these elements on technology acceptance, future research and app development should integrate strategies that address behavior change to enhance user engagement and long-term adoption. It is also important to consider that the effectiveness of an intervention is not solely determined by the number of BCTs used, but rather by their relevance and alignment with the behavioral change needs and characteristics of the target population.

Moreover, the findings of this review suggest that the combination of BCTs and gamification elements may play a key role in supporting adherence and sustained engagement in digital interventions for UI. Core BCTs such as goal-setting, self-monitoring, feedback, and guided repetition function as psychological mechanisms that facilitate habit formation and long-term behavioral maintenance [17], while gamification elements can amplify these effects by enhancing intrinsic motivation, perceived competence, and the visibility of progress [13]. This interaction is particularly relevant for older adults, who often require additional motivational support to maintain behavioral routines over time [11]. However, the variability observed in the selection and implementation of BCTs across the included apps highlights the need for future digital interventions to adopt more explicit theoretical frameworks and a systematic selection of BCTs aligned with the behavioral needs of people with UI.

Several BCTs appear particularly relevant for UI management. Social support can reduce stigma and feelings of isolation, thereby increasing motivation and adherence to pelvic floor muscle training. Regulation strategies, such as self-monitoring of symptoms and fluid intake, enhance patients’ sense of control and facilitate consistent practice. Antecedents, including the identification and modification of triggers (eg, caffeine consumption or timing of fluid intake), help minimize risk situations and improve symptom management. Strengthening identity by fostering the role of the patient as an active self-manager promotes internalization of healthy routines and long-term commitment [57]. The use of scheduled consequences, such as rewards or positive feedback for adherence to exercises, reinforces behavior through operant conditioning. Finally, covert learning techniques, including guided imagery or mental rehearsal, allow patients to visualize successful execution of exercises and coping strategies, which can increase confidence and improve real-world performance. Together, these BCTs target mechanisms of motivation, self-efficacy, and habit formation, thereby supporting adherence and sustainable behavior change in UI interventions.

The gamification elements identified in the apps were limited, although all apps include some gamification element. URinControl and Tät still follow app models with drawings instead of images, while BladderBoss used real-life pictures and videos. In all the apps, exercises are classified by levels, and the user progresses from one level to another. For example, in URinControl, users receive a medal upon completing a level.

All the apps offer the option to view the evolution or progress of the exercises and urine leakage. None incorporate a game within the app or allow users to rate the exercises, receiving a score upon completion, view user ranking, or earn rewards, among other gamification elements.

Several of the studies reviewed provided a qualitative assessment of app use through interviews. These analyses identified that adherence directly influences the outcomes of the application, acting both as a barrier (eg, difficulty in finding the time to perform the exercises) and as a facilitator. Adherence itself was shaped by personal factors, app-related characteristics, and users’ awareness. A frequently reported concern was the insecurity experienced by users when performing the exercises, as many were unsure whether they were executing them correctly.

On the positive side, the novelty of technology in this type of treatment was highlighted, together with its accessibility; patients do not need to wait on long lists to receive care, and exercises are always available. The apps were also valued as practical guides that support users in performing the exercises while preserving privacy. Many participants emphasized that the use of apps increased their awareness of symptoms and improved their education about the condition.

The scientific literature consistently supports the effectiveness of mobile apps in enhancing urinary continence, improving quality of life, and increasing patients’ perception of symptomatic relief. However, variability in statistical significance was observed across studies. Specifically, interventions using the Tät [32,40-42] and Tät II [31,33,34] apps demonstrated statistically significant differences between groups, whereas URinControl [35,37,39] showed improvements that were not statistically significant.

Treatment outcomes were influenced by several factors, including users’ adherence, personality traits, integration of exercises into daily routines, and previous experience with pelvic floor training. Conversely, the primary barriers identified were unrealistic patient expectations and individual difficulties in maintaining adherence.

These findings highlight the necessity of optimizing digital interventions to ensure greater personalization and clinical effectiveness. Key determinants such as symptom severity, age, educational background, and the impact of UI on quality of life should be integrated into the design of eHealth interventions to enhance their predictive validity and therapeutic outcomes. Overall, the results emphasize the importance of developing mobile apps tailored to the specific needs of the target patient population to enhance adherence, improve usability, and ultimately contribute to better symptom management and quality of life.

In summary, although few evidence-based apps or websites are available for people aged 50 and over, existing tools suggest potential benefits in reducing UI and improving quality of life. However, the mixed statistical significance across studies underscores the need for further research to confirm these effects. Moreover, this review highlights the importance of personalizing digital interventions to maximize their effectiveness and ensuring that future apps incorporate a broader range of BCTs and more robust gamification strategies. Further studies are needed to verify the effectiveness of these apps and to expand the availability of high-quality, evidence-based tools specifically designed for this population

### Limitations and Strengths

One of the main limitations of this study is that the review was conducted in only three countries, which may restrict the generalizability of our findings. Additionally, some apps were not available on the App Store, and we did not search all app markets (eg, Amazon or the Xiaomi App Store), leading to potential gaps in our evaluation. This limitation may have influenced our ability to provide a fully comprehensive analysis of all relevant apps in the field.

The study was limited by the absence of a structured instrument to objectively assess the presence of gamification in the different apps. Nevertheless, the development of such a tool holds considerable potential for future research and could contribute to more precise and standardized evaluations.

Despite these limitations, our study has several key strengths. A rigorous methodology was applied, ensuring the reliability of the findings, and the evaluation process was conducted in duplicate, involving several independent reviewers, which enhanced objectivity. Furthermore, all included apps were thoroughly assessed using the MARS [29] and BCT frameworks, providing a robust framework for evaluation. Additionally, in cases where no clear scientific evidence was available regarding an app’s effectiveness, we proactively contacted the respective companies to inquire about any supporting research papers. This approach reinforced the credibility and transparency of our findings.

## Supplementary material

10.2196/76867 Checklist 1 PRISMA 2020 checklist.

## Abbreviations

BCT: behavior change technique
ICIQ-OAB: International Consultation on Incontinence Questionnaire - Overactive Bladder
ICIQ-SF: International Consultation on Incontinence Questionnaire - Short Form
MARS: Mobile Application Rating Scale
ORCHA: Organisation for the Review of Care and Health Apps
PA: physical activity
PGI-I: Patient Global Impression of Improvement
PICO: Population, Intervention, Comparison, and Outcome
PMPT: Pelvic Muscle Physical Therapy
UI: urinary incontinence
